# Relationship to pain and suicidal-related experience: Validation of discomfort intolerance scale и the pain catastrophizing scale in russian female adolescents

**DOI:** 10.1192/j.eurpsy.2021.1572

**Published:** 2021-08-13

**Authors:** V. Sadovnichaja, E. Rasskazova, A. Tkhostov

**Affiliations:** 1 Clinical Psychology, Moscow State University, Moscow, Russian Federation; 2 Department Of Neuro- And Pathopsychology, Faculty Of Psychology, Lomonosov Moscow State University, Moscow, Russian Federation

**Keywords:** relationship to pain, discomfort intolerance scale, the pain catastrophizing scale

## Abstract

**Introduction:**

Perception of and relationship to pain are considered as important factors of suicidal behavior (Joiner, 2005, Klonsky & May, 2015, O’Connor & Kirtley, 2018, Galynker, 2017). Some studies of pain demonstrated that there are common mechanisms of emotional and physical pain (DeWall & Baumeister, 2006, MacDonald & Leary, 2005, Eisenberger, Lieberman, & Williams, 2003).

**Objectives:**

The aim was to validate Discomfort Intolerance Scale and Pain Catastrophizing Scale on the female adolescent sample and to reveal their relationship to suicidal experience.

**Methods:**

183 adolescents females (13-21 years old) filled Discomfort Intolerance Scale (Schmidt, Richey, & Fitzpatrick, 2006) and The Pain Catastrophizing Scale (Sullivan, Bishop, & Pivik, 1995). Then they replied to items related to their own or their friends’ suicidal experience.

**Results:**

Factor analysis for PCS explained 73.6% of variance with Cronbach’s alphas .77-.91. Factor analysis of DIS explained 67.1% of variance with Cronbach’s alphas .63-.70. There were no relationships between suicidal-related experience and pain-related experience.

**Conclusions:**

Discomfort Intolerance Scale and Pain Catastrophizing Scale could be used as reliable and valid methods of measuring relationship to pain in studies of adolescents, although we found no associations between them and suicidal intentions.

